# Prioritizing novel and existing ambulance performance measures through expert and lay consensus: A three‐stage multimethod consensus study

**DOI:** 10.1111/hex.12610

**Published:** 2017-08-25

**Authors:** Joanne E. Coster, Andy D. Irving, Janette K. Turner, Viet‐Hai Phung, Aloysius N. Siriwardena

**Affiliations:** ^1^ University of Sheffield Sheffield UK; ^2^ Community and Health Research Unit University Lincoln Lincoln UK

**Keywords:** ambulance, consensus methods, delphi, outcome measurement, patient and public involvement, quality and performance

## Abstract

**Background:**

Current ambulance quality and performance measures, such as response times, do not reflect the wider scope of care that services now provide. Using a three‐stage consensus process, we aimed to identify new ways of measuring ambulance service quality and performance that represent service provider and public perspectives.

**Design:**

A multistakeholder consensus event, modified Delphi study, and patient and public consensus workshop.

**Setting and participants:**

Representatives from ambulance services, patient and public involvement (PPI) groups, emergency care clinical academics, commissioners and policymakers.

**Results:**

Nine measures/principles were highly prioritized by >75% of consensus event participants, including measures relating to pain, patient experience, accuracy of dispatch decisions and patient safety. Twenty experts participated in two Delphi rounds to further refine and prioritize measures; 20 measures in three domains scored ≥8/9, indicating good consensus, including proportion of calls correctly prioritized, time to definitive care and measures related to pain. Eighteen patient/public representatives attended a consensus workshop, and six measures were identified as important. These include time to definitive care, response time, reduction in pain scores, calls correctly prioritized to appropriate levels of response and survival to hospital discharge for treatable emergency conditions.

**Conclusions:**

Using consensus methods, we identified a shortlist of ambulance outcome and performance measures that are important to ambulance clinicians and service providers, service users, commissioners, and clinical academics, reflecting current pre‐hospital ambulance care and services. The measures can potentially be used to assess pre‐hospital quality or performance over time, with most calculated using routinely available data.

## 1 INTRODUCTION

### 1.1 Background

Ambulance services are increasingly providing front‐line care for a wide range of patients with emergency and urgent conditions, which in the past were the domain of primary care or emergency departments (ED).^1^ The widening scope of practice of ambulance services and clinicians means that reliance on conventional measures of ambulance care, such as response times, does not adequately represent the range of patient conditions or different types clinical management in the pre‐hospital environment and is inadequate for measuring service performance and quality.^2^ Although new measures of performance and quality have been promoted,^3^ developed and applied,^4^, ^5^ international comparisons of pre‐hospital Emergency Medical System (EMS) performance indicators show that measures have only been developed for a limited range of conditions,^6^ and research to inform the development of wider measures is a recognized priority.^7^ Prior research has largely focussed on developing measures for emergency medicine and urgent care systems rather than pre‐hospital ambulance services.^8^, ^9^ There is also very little known about which measures members of the public find meaningful or important.

### 1.2 Importance

Ambulance services have limited scope to measure the quality and performance of their services due to an absence of information about what happens to patients after ambulance discharge and a lack of consensus about which outcomes are important as measures of good‐quality care. Without the identification and development of measures related to current practice that reflect the whole ambulance service, there is little opportunity for identifying problems of care delivery, good practice or evaluating service developments.

Quality measurement and improvement are a recognized priority for health services due to increasing public demand, consumerism, scientific evidence for new treatments and political pressure arising from failures in care quality.^10^ This necessitates the development of better quality measures, particularly for ambulance services where the nature of provision is changing rapidly. Changes have been driven by multiple factors including new and existing health technologies;^11^ advances in education and training of clinicians including developments such as advanced paramedic practitioners with an enhanced scope of practice;^12^ and policy changes that have encouraged more ambulance treatment and care outside hospital.^1^ In England, current ambulance quality indicators (AQIs) have developed from previous time‐based targets to include service process and clinical indicators, but these are condition‐specific and predominantly relate to patients with high urgency conditions.^5^ Given that fewer than 10% of ambulance calls are for life‐threatening problems, it is important that measures relating to the whole ambulance population are developed.^4^

### 1.3 PhOEBE research programme

The Pre‐hospital Outcomes for Evidence Based Evaluation (PhOEBE) project is a 5‐year NIHR research programme which aims to develop new ways of measuring the quality, performance and impact of pre‐hospital care provided by ambulance services. The research aims to address the dual problems of ambulance services’ poor access to patient information post‐discharge and lack of consensus about what are good ambulance service quality measures.

### 1.4 Goals of this investigation

We aimed to identify, refine and prioritize a set of quality and performance measures that are important to patients and the public, ambulance service care providers and the wider pre‐hospital community. Such measures could be used to assess care quality over time both within and between services and to support audit, quality improvement and research by measuring the impact of improvements and innovations in ambulance service care.

## 2 METHODS

### 2.1 Study design

We conducted a three‐stage multimethod consensus study: Stage 1 modified nominal group technique (NGT) multistakeholder consensus event; Stage 2 Modified Delphi study; Stage 3 Patient and Public Involvement (PPI) consensus workshop. This iterative approach allowed the gradual refinement of a long list of potential candidate measures down to a smaller number for further development and to reflect a range of perspectives. Due to the large number of measures identified from the literature, the Delphi stage was preceded by a consensus event to undertake first‐stage prioritization and sifting to ensure the feasibility of the Delphi study. PPI concerns over the suitability of the Delphi method for PPI participants resulted in a separate PPI consensus event.

### 2.2 Indicators or measures

When selecting types of indicator, it is important to consider whether they are in fact indicators or measures as the terms can be used interchangeably. Indicators are by their very nature indicative of performance and quality, but are not direct measures of it. For this study, measures were preferable to indicators as we wished to measure service performance. However, we also included some service‐specific measures which were considered to be indicators of performance.

### 2.3 Candidate measures

The study team undertook two systematic literature reviews. Review 1 focussed on policy reports to identify actual and aspirational measures of ambulance performance, and used a systematic approach to identify relevant documents. Review 2 was a systematic search and synthesis of performance and outcome measures reported in published pre‐hospital care research.^13^ By identifying what could or should be measured and also what was currently being measured, we generated a list of potential measures to prioritize and refine using consensus methods. Recognizing the predominance of process measures reported in the literature, and to ensure patient and service user views were included, we undertook interviews with recent users of the ambulance service to find out what mattered to them.^14^ We also held a focus group with patients and members of the public specifically to identify any additional aspects of ambulance service care that are considered important. From these, we developed a broad list of 72 measures, of which 29 were time‐based measures. Where measures were identified from policy documents or patient interviews, these sometimes related to important principles rather than a defined measure, for example, measuring patient safety or patient experience.

### 2.4 Stage 1: Modified nominal group technique consensus event

#### 2.4.1 Recruitment and participants

Consensus event participants were recruited by inviting representatives from all UK ambulance services, professional groups, including the National Ambulance Research Steering Group (NARSG), Association of Ambulance Chief Executives (AACE), National Ambulance Service Clinical Quality Group (NASCQG), National Ambulance Commissioning Group (NACG), College of Paramedics and College of Emergency Medicine (CEM). We also invited PPI representatives from the PhOEBE research programme reference group and members of the Sheffield Emergency Care Forum (SECF) PPI group. The SECF PPI group cascaded the invitation to other PPI groups representing emergency and urgent care. Service commissioners (those responsible for planning/purchasing NHS services to meet local population health needs), policymakers and clinical academic emergency medicine representatives were also invited to attend. Potential participants initially registered their interest in attending the consensus event, with the opportunity to confirm this nearer to the time. No patients were recruited from within the NHS.

#### 2.4.2 Categorization of measures

We categorized candidate measures into three groups: (i) ambulance service activities and operations (n=14); (ii) direct clinical management of patients (n=20); and (iii) impact of care on patients (n=9), based on a Donabedian approach of structure, process and outcome.^15^ Due to the large number of time measures identified (n=29), these were excluded from the three groups, to avoid an over emphasis on time‐based process measures during the group discussions. Time measures were sent out in an online format for consideration prior to the event. Therefore, the measures discussed in the small groups were the 43 non‐time measures, but both these and the time measures were then presented for voting.

The Donabedian model was chosen to ensure a balance of measures that represented the full range of ambulance service activities, and also because it is a widely used conceptual model that is easily communicated to and understood by research participants.^15^ The full list of measures is provided in Appendix S1.

#### 2.4.3 Prioritization of measures

We used a modified NGT to prioritize and rank measures. NGT is a structured group meeting of experts with the process led by a moderator.^16^ This approach allows face‐to‐face interaction and discussion between participants, which is crucial at the early consensus stage. The NGT was modified to incorporate electronic voting and to include our identified candidate measures as a starting point for group discussions. We held small group discussions for each group of measures, facilitated by members of the research team. Participants were encouraged to think of additional measures to share with the group, using a round robin format, ensuring each participant had an opportunity to contribute. Discussion sessions were immediately followed by voting to rank the importance of each measure or principle as a potential measure of good‐quality ambulance service care. Participants voted using an anonymous audience response voting system (Turning Technologies, Youngstown, OH, USA)^17^ and were asked to decide whether each measure was essential, desirable or irrelevant by pressing a single button on a handset. The list of 29 time‐based measures was also presented for voting using the same criteria.

### 2.5 Stage 2: Modified Delphi study

#### 2.5.1 Questionnaire development

The consensus event was concerned with identifying what was important to measure, whereas the modified Delphi study was concerned with how this could be measured. This was particularly important for hard to measure concepts and principles that were included in Stage 1. We developed an electronic modified Delphi questionnaire by including measures from the consensus event that were rated as essential or desirable, or that were highly rated by PPI attendees. Therefore, a primary function of Stage 1 was to decide what to exclude from subsequent consensus stages rather than only focussing on what to include. Delphi measures were categorized into three groups, again based on the Donabedian framework:^15^ whole service measures (structure) (n=32); clinical management measures (process) (n=10); and patient outcomes (outcome) (n=25). The number of measures was higher than those considered in the consensus event, as at this stage, we included time measures and began to develop more explicit, discrete descriptions of potential measures. For example, where a broad principle such as accuracy of dispatch decisions was used for the consensus event, this was developed as multiple possible measures derived from the consensus event discussions in relation to specific conditions or call types. Participants were asked to consider each measure and score their level of agreement on a scale of 1‐9 (strongly disagree to strongly agree) using the statement:

*This measure (either on its own or within a set of measures) is a good reflection of the quality of care provided by ambulance services and is likely to be a good indicator of the quality of the 999 ambulance service care pathway*.

We asked participants not to consider the current availability and quality of, or difficulties in access to relevant data when scoring the measures, to allow novel measures to be included. Participants were able to suggest additional measures for inclusion using a free text box.

#### 2.5.2 Recruitment and participants

Stage 1 expert participants were asked whether they would like to participate in Stage 2. We also recruited additional Delphi participants through targeted emails to specific individuals known to be experts in fields related to ambulance service care or care delivery. PPI participants were not included in the Delphi because our PPI reference group felt the Delphi method was not suitable for PPI participants because of the complexity of the topic. We sought advice from our PPI reference group and other PPI experts on how best to involve service users and this is reported in Stage 3.

Participants included senior paramedics and operational staff, ambulance medical directors, research and audit staff, members of the NARSG and NASCQG, commissioners, emergency care physicians and academics.

#### 2.5.3 Delphi process

We followed a RAND‐based Delphi approach, whereby “a group of experts who anonymously reply to questionnaires and subsequently receive feedback in the form of a statistical representation of the ‘group response’, after which the process repeats itself”.^18^ In round 1 of the Delphi process, participants scored each measure, gave text comments and suggested additional measures or revisions to existing measures, where appropriate. In round 2, we provided each participant with their individual score, the median group score for each measure, any text comments from the previous round and a small number of additional measures/revisions to the wording of measures based on round 1 comments. For the second round, we asked participants to consider their original score for each measure in the light of the median score of the group and the participant comments. Up to two reminders were sent unless participants indicated they no longer wished to take part.

### 2.6 Stage 3: Patient and public involvement consensus workshop

Our study PPI reference group felt the Delphi exercise contained too much technical information for patient and public representatives to participate meaningfully and that the complexity of some concepts and measures would be better explained and discussed in a face‐to‐face format. Therefore, we held a separate face‐to‐face PPI workshop to increase opportunities for meaningful PPI engagement with technical, complex and often little known aspects of ambulance service performance. The detailed study methodology is reported as a separate paper, but the results are integrated into this analysis.^19^

#### 2.6.1 Recruitment and participation

Stage 3 PPI participants were recruited via local PPI networks. Other participant groups were not included at this stage as their involvement occurred as part of Stage 2. A wide range of PPI groups were targeted, including vulnerable and hard to reach groups.

#### 2.6.2 Analysis

Stage 1 consensus event results were analysed using SPSS version 21 (IBM, Armonk, New York, USA). We identified the number and proportion of essential, desirable and irrelevant votes for each measure. We ranked the results by the proportion of essential and irrelevant votes to identify measures with the most and least agreement.

Stage 2 Delphi responses to round 1 and round 2 were entered into SPSS version 21. The median score for each measure was calculated because Delphi techniques incline scores towards middle values. We also calculated the change in median scores between rounds 1 and 2. As there was very little score change between the rounds, we considered a third round unnecessary. We ranked measures by their median scores to classify whether measures achieved a “good,” “moderate” or “poor” level of consensus, which is a commonly used definition for consensus.^20^ A low score (negative consensus) threshold was identified as a score of 5 or less. Measures were retained for inclusion in the PPI consensus workshop if they achieved moderate or good consensus, or had previously been identified as important by PPI participants and were considered as measurable using routinely collected data. This was broader than the usual RAND criteria^18^ because the PPI workshop was considered a parallel process to the Delphi study rather than a subsequent stage. We wanted patient and public views on a wide range of measures and not just those that had achieved good consensus from the Delphi participants.

The proportion of PPI votes for each measure was identified in Stage 3, and these considered are alongside the Delphi results.

#### 2.6.3 Integration of results

To achieve a final list of measures, we convened a small expert group to consider which measures should be further developed as part of the PhOEBE research programme. Because services have many components, we aimed to select a set of measures that represented and assessed the quality of a service. Measures were considered against the following attributes: importance and relevance; validity (evidence based); measurable using the PhOEBE data set; simple to understand; remediable (the ambulance service can influence performance). A shortlist of eight measures was selected for development (see Table 1).

**Table 1:** Final set of measures

Measure description	Aim
Change in pain score (mean/median)	To calculate the change in pain score for patients who received an ambulance response and had more than one pain score recorded
Accuracy and appropriateness of call ID	To identify the proportion of patients with serious emergency conditions whose condition is appropriately categorized by the ambulance service
Average response time	To calculate the average ambulance response time for an ambulance service (median)
Proportion of decisions to leave a patient at scene (hear and treat and see and treat) which resulted in recontacts and/or death (within 3 d)	To identify the frequency of potentially inappropriate non‐conveyance decisions
Proportion of ambulance patients with a serious emergency condition who survive to admission, and to 7 d post‐admission	To identify the proportion of people with a serious emergency condition who survive to admission (within 7 d of ambulance contact), and of those, the proportion who survive to 7 d post‐admission
Proportion of ambulance service contacts for patients with specific, urgent health problems presenting a low risk of death, where the patient subsequently died from such a cause within 3 d	To identify the proportion of people who died and were at a low risk of dying
Proportion of patients transported to ED by 999 emergency ambulance who were discharged to usual place of residence or care of GP, without treatment or investigation(s) that needed hospital facilities	To identify the frequency of potentially inappropriate conveyance decisions
Proportion of all cases with a specific condition who are treated in accordance with established protocols and guidelines, for example stroke, heart attack, diabetes, falls	To identify the proportion of patients who are treated according to defined care pathways

## 3 RESULTS

### 3.1 Stage 1: Modified nominal group technique consensus event

**Figure 1:**
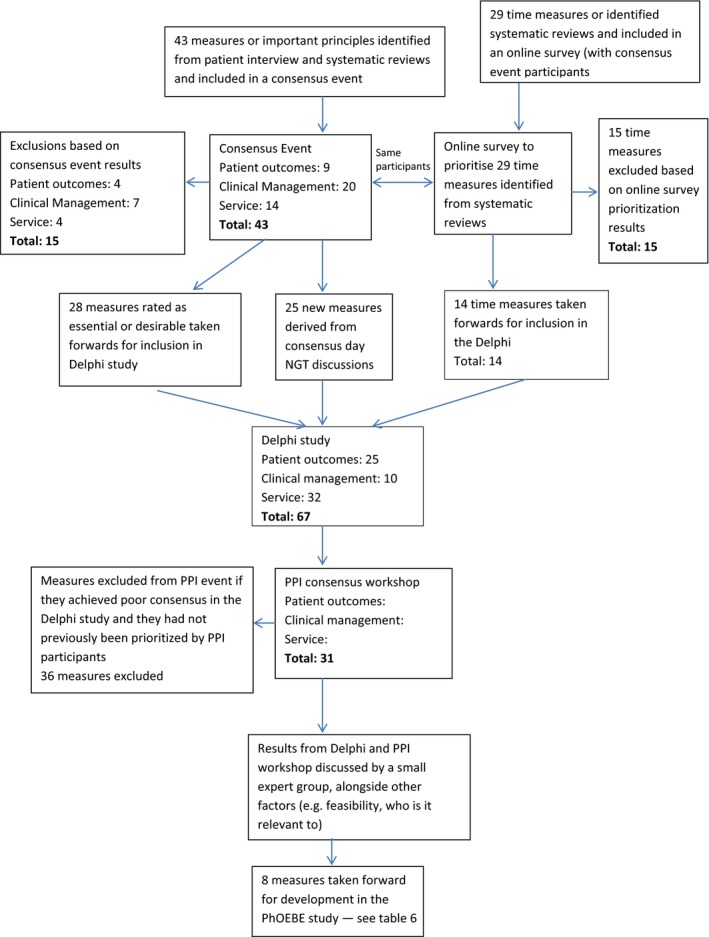
Consensus flow chart

From 63 people who expressed an interest in attending the consensus event, 42 (67%) attended. Most participants were UK‐based and from a range of locations. We had international representation from the USA, Australia and Denmark as quality in ambulance service performance is an international issue that other countries are also trying to resolve. Eleven of the participants represented PPI groups. The remaining participants represented ambulance services, emergency medicine, clinical research and ambulance strategy and commissioning. A full list of the job titles of attendees is available as Appendix S1 and number of participants approached and recruited in Table 2. Eight of the 11 regional English Ambulance Services were represented at this event to consider 43 measures (Figure 1).

**Table 2:** Participants included at each stage

Stage/Event type	Participant group							Total
Stage 1: Consensus event	PPI	Ambulance clinical	Ambulance operations management	Commissioners	Policy makers	Emergency care	Academic	
Number of people who registered an interest in attending	13	14	15	5	4	7	5	63
Total number of people who attended	11	9	8	4	3	4	4	43
Stage 2: Delphi study	PPI	Ambulance clinical	Ambulance operations management	Commissioners	Policy makers	Emergency care	Academic	
Total number approached								
Total number participants agreed to participate	Not approached to participate due to PPI reference groups preference for face‐to‐face involvement	7	8	3	0	3	(emergency care participants were also academics)	21
Total participate in round 1	0	7	8	3	0	3	N/A	21
Of these, total number who participate in round 2	0	7	7	3	0	3	N/A	20
Stage 3: PPI workshop	PPI	Ambulance clinical	Ambulance operations management	Commissioners	Policy makers	Emergency care	Academic	PPI
Total number registered to attend	19	This event was specifically for PPI participants to obtain their views about the Delphi items. No other participant groups were asked to attend						19
Total number attended	18	0	0	0	0	0	0	18

#### 3.1.1 Response rates

For 16 of the 42 votes, the response rate was 100%. Slight fluctuations in voting were due to a small number of participants not able to attend the full event. The response rate was consistently high, with the lowest being 39/42 (93%).

#### 3.1.2 Key results

The 10 highest ranked outcome measures/measurement principles are shown in Table 3, ranked according to the percentage rated “essential.” Most participants (69%‐86%) rated these measures as essential, and most were rated highly by all participant groups. High ranking measures focussed on decision making (eg. accuracy of dispatch and triage decisions, appropriateness of service provided), compliance with protocols and guidelines (including end of life care plans), patient safety and pain relief.

**Table 3:** Highest ranked measures according to percentage rated essential in modified nominal group consensus event

Rank	Measure	Essential (E) n (%)	Desirable (D) n (%)	In favour (E+D) n (%)	Irrelevant n (%)	Total votes
1	Accuracy of dispatch decisions	36 (86)	6 (14)	42 (100)	0	42
2	Completeness and accuracy of patient records	35 (85)	5 (12)	40 (97%)	1 (2)	41
3	Accuracy of call taker identification of different conditions or needs	33 (79)	7 (17)	40 (96%)	2 (5)	42
4	Pain measurement and symptom relief	33 (79)	7 (17)	40 (96%)	2 (5)	42
5	Patient experience	31 (78)	9 (22)	40 (100%)	0	40
6	Measuring patient safety	32 (76)	9 (22)	41 (98%)	1 (2)	42
7	Over—triage rates and under triage rates	31 (76)	9 (22)	40 (98%)	1 (2)	41
8	Compliance with end of life care plans	31 (76)	7 (17)	38 (93%)	3 (7)	41
9	Proportion of calls treated by most appropriate service	30 (75)	9 (23)	39 (98%)	1 (2)	40
10	Compliance with protocols and guidelines	29 (69)	12 (29)	41 (98%)	1 (2)	42

Low ranked measures tended to be further along the care pathway, had greater potential to be influenced by multiple care providers, or only related to a small proportion of the ambulance population, for example duration of inpatient life support, length of hospital stay or proportion of people receiving spinal immobilization for back/neck injuries. These results informed the subsequent Delphi study.

### 3.2 Stage 2: Delphi study

In all, 23 Delphi participants from round 1 and 20 from round 2 returned completed questionnaires (see Table 2). The overall response rate, based on participants who completed both rounds, was 74 per cent. Participants represented wide‐ranging service provider and professional viewpoints, and most UK ambulance trusts.

Most measures scored highly in the Delphi study, with 66% (40/61) of measures scoring 7 or above. Based on the data distribution, high scores were defined as 8 and above (Table 4), rather than our a priori high score of 7 based on previous research.^9^ This was due to the large number of measures scoring 7 or above rendering the a priori high score ineffective at discriminating between measures. Basing the high score threshold on the data distribution resulted in 30% (20/67) of measures achieving a high score. No measures scored less than 4. There was little change in the scores given by participants between rounds. Scores for most items remained stable between the rounds; a small number of items had a score change of +0.5 or −0.5. This negated the need for a third round as consensus had been achieved.

**Table 4:** Classification of high and low scores

Consensus	Median score	Measures (n)
Good	≥8 (high)	20
Moderate	6–7 (medium)	36
Poor	<6 (low)	11

### 3.3 Stage 3: PPI workshop

Eighteen PPI representatives attended the PPI workshop exemplifying a range of people, including young people and vulnerable groups.

### 3.4 Stage 2 and 3 key results

Delphi and PPI workshop results are presented by category of measure (Tables 5, 6, 7). Low scoring Delphi measures (<6) and measures excluded from the PPI event for other reasons, for example not currently measurable using routine data, were excluded from these tables (see Table S1).

**Table 5:** Delphi and PPI results: patient outcome measures

ID	Patient outcome measures	Delphi score Median; IQR (Range)	PPI Vote n (%)
PO1b	Proportion of patients who report pain who are given analgesia (pain relief)	8; 7–8 (1–9)	1 (5.5)
PO3a	Proportion of patients with cardiac arrest where resuscitation is attempted at the incident scene who have a pulse on arrival at the emergency department	8; 4.5–9 (1–9)	2 (11)
PO6b	Proportion of all 999 calls referred for telephone advice only recontacting the ambulance service within 24 h	8; 7.25–9 (5–9)	2 (11)
PO1a	Proportion of all patients seen by an ambulance crew who have a pain assessment recorded	7; 7–8 (1–9)	4 (22)
PO1c	Proportion of patients who have a reduction in pain score after analgesia treatment	7; 6–8.75 (3–9)	9 (50)
PO1d	Proportion of patients reporting pain who have more than one pain score recorded	7; 7–8 (3–9)	4 (22)
PO2c	Proportion of patients who report that key aspects of care were delivered. (examples of key aspects are timeliness of response; reassurance; professionalism; communication; smooth transition between/within services	7; 6–7.75 (3–9)	Did not vote^a^
PO5c	Proportion of patients with a life‐threatening condition (amenable to emergency treatment) who are discharged alive from hospital	7; 5–7.5 (1–9)	11 (61)
PO6a	Proportion of all 999 calls recontacting the ambulance service within 24 h	7; 6.25–8 (3–9)	8 (44)
PO6e	Proportion of patients left at home who are admitted to hospital within 72 h	7; 7–8 (1–9)	2 (11)
PO5a_1	Proportion of 999 callers who die within 0‐48 h of first call	6; 4–7 (1–9)	5 (28)
PO6c	Proportion of patients left at home who have a contact with any emergency/urgent health service within 24 h	6; 5.25–7.75 (3–9)	6 (33)

a PPI participants felt this measure was too broad to vote on.

**Table 6:** Clinical Management measures

ID	Clinical Management measures	Delphi score Median; IQR (Range)	PPI Vote n (%)
CM1a	Proportion of all calls referred for telephone advice returned for a 999 ambulance response	8; 6–8.75 (3–9)	2 (11)
CM1b	Number of calls prioritized correctly to appropriate level of response as a proportion of all 999 calls	8; 7–8.75 (1–9)	12 (67)
CM1c	Proportion of life‐threatening category A calls correctly identified as category A	8; 8–9 (6–9)	3 (17)
CM2a	Proportion of all cases with a specific condition who are treated in accordance with established protocols and guidelines, for example stroke, heart attack, diabetes, falls	8; 8–9 (6–9)	12 (67)
CM2b	Proportion of cases that comply with end of life care plans where these are available	8; 8–8.75 (6–9)	0 (0)
CM2c	Proportion of all cases with a specific condition who meet established criteria for transfer, who are transported to an appropriate specialist facility, for example a heart attack, stroke or major trauma centre	8; 8–9 (2–9)	6 (33)
CM1d	Proportion of calls for specific condition correctly identified at during the call, for example cardiac arrest, stroke, heart attack	7; 7–9 (3–9)	1 (5.6)

**Table 7:** Whole system measures

ID	Whole system measures	Delphi score Median; IQR (Range)	PPI Vote n (%)
WS6d	Time of call to CPR start time (if CPR is required); average time from call to start of CPR in cases of cardiac arrest	9; 8.25–9 (7–9)	0 (0)
WS6e_1	Proportion of eligible calls who arrive at definitive care within agreed timescales, for example at a specialist heart attack centre within 150 min	9; 9–9 (8–9)	9 (50)
R2_WS6a_2_30min	Proportion of emergency calls for conditions that are not life‐threatening with a response time of 30 min or less	9; 8–9 (7–9)	2 (11)
WS6a_1	Proportion of emergency calls with a response time within an agreed standard for calls for life‐threatening conditions	8; 7–9 (4– 9)	2 (11)
WS6e	Time of call to time to definitive care	8; 8–9 (6–9)	9 (50)
WS2a	Number of life‐threatening (category A) calls not identified as category A as a proportion of all 999 calls	7; 7–8 (3–9)	3 (17)
WS2b	Number of calls that are not life‐threatening identified as category A calls as a proportion of all 999 calls	7; 5.25–7 (1–9)	1 (5.6)
WS3b	Proportion of category A calls attended by a paramedic	7; 6.25–8 (1–9)	5 (28)
WS3c	Proportion of patients treated on scene or left at home who are referred to appropriate pathways (primary care)	7; 7–8 (3–9)	5 (28)
WS3e	Proportion of patients transported to ED by 999 emergency ambulance and discharged without treatment or investigation(s) that needed hospital facilities	7; 6–8.75 (3–9)	1 (5.5)
WS3f	Proportion of patients who potentially could be left at home who are successfully discharged at the scene.	7; 6–7 (1–9)	3 (17)
WS6a	Time of call to time of arrival at scene/Proportion of emergency calls with response times within agreed standards	7; 6.25–7.75 (3–8)	14 (78)

#### 3.4.1 Patient outcome measures

Fourteen of seventeen patient outcome measures that achieved moderate to high scores in the Delphi were considered at the PPI event (Table 5). The three measures not considered by PPI had poor consensus in the Delphi study, were condition‐specific or were not considered important by our PPI reference group. Measures identified as most important from the PPI event were proportion of patients with a life‐threatening condition (amenable to emergency treatment) who are discharged alive from hospital and proportion of patients who have a reduction in pain score after analgesia treatment. These both achieved moderate consensus from the Delphi study. Recontacts, for example the proportion of people receiving telephone advice who contact the ambulance service within 24 hours, were highly prioritized by Delphi participants.

#### 3.4.2 Clinical management

The clinical management category contained the fewest number of measures; therefore, all seven measures were considered by Delphi participants and PPI participants (Table 6). Nearly, all measures were highly scored by the Delphi participants (6/7 measures) and two measures were also identified as important by PPI participants. These were as follows: number of calls prioritized correctly to appropriate level of response as a proportion of all 999 calls and proportion of all cases with a specific condition who are treated in accordance with established protocols and guidelines, for example stroke, heart attack, diabetes and falls.

#### 3.4.3 Whole system

This category contained the most measures (n=24) and was also the highest scoring category in the Delphi study (Table 7). Eleven of these measures were presented at the PPI consensus workshop. Many of the measures related to time standards or time to definitive care for particular conditions. As PPI participants were unable to prioritize one clinical condition over another (with all being considered equally worthy), these were considered by PPI participants as a single measure for all conditions (see Table S1 for the full list of excluded measures). Time to definitive care achieved a score of 9 in the Delphi and was identified as important by 50% of PPI participants. Time of call to time of arrival at scene/Proportion of emergency calls with response times within agreed standards was also scored highly by PPI participants (78%), but only achieved moderate consensus from the Delphi participants.

## 4 DISCUSSION

### 4.1 Main findings

Using a three‐stage consensus process, we prioritized a set of ambulance outcome and performance measures. These measures reflected current pre‐hospital ambulance care and services and were important to ambulance clinicians, service providers, commissioners, clinical academics and PPI. The measures represented key concepts and principles, such as patient safety and triage accuracy. Most of the prioritized measures can be calculated with available routine data.

#### 4.1.1 Comparison with other literature

Measuring a single outcome or aspect of a service can give an incomplete assessment of the quality of care within an organization and can be a misleading guide to the overall performance of an organization,^21^whereas using a broader range of measures is more likely to reflect the complexity and range of care provided.^22^ For many years, ambulance services internationally have focussed on time measures, particularly response time performance as a primary target, while patient and clinical outcomes were not considered.^6^, ^23^ A response time target, such as the 8‐minute ambulance response for immediately life‐threatening calls used in the UK, is based on the need for a fast response to people with out‐of‐hospital cardiac arrest, but these only account for under 5% of ambulance service workload. A study into paramedic views of response times found they were considered inadequate performance measures, being simplistic, narrow and only covering one aspect of the patient care pathway.^23^ There was also concern that time targets might distort clinical priorities. The results from the study by Price and colleagues advocated measures that recognize the full patient pathway, from the initial call for help to safe discharge (to home or to hospital) from the ambulance service.^23^ Furthermore, it is acknowledged by a number of studies that a set of measures that together reflect a whole service is a better approach for quality and performance measurement.^24^, ^25^ McClelland argued that an “intelligent suite of targets which incentivize change and provide a greater focus on patient experience and outcomes” was required.^24^ Martin et al.^25^ supported a whole system outcome measurement method in which a balanced set of measures was used. Time measures cannot be excluded, as our study found that response times were considered important. Nevertheless, other methods of measurement, for example average response time, should be explored.

In England, the assessment of ambulance service performance and quality has evolved from single response time‐based measures to a set of indicators introduced in 2011, which also included clinical processes and intermediate outcomes to drive improvements in care.^4^, ^26^ As well as response time and survival from out‐of‐hospital cardiac arrest, this broader range of measures comprised clinical management indicators for specific conditions such as cardiac arrest, myocardial infarction and stroke, and additional broader process measures including recontact rates within 24 hours for patients who received telephone advice or who were treated at scene. While this went some way to addressing the lack of information about the quality of ambulance service care, the clinical measures were condition‐specific, process‐focussed and mainly applied to a small proportion of the most critically ill patients rather than the whole population using the service.

### 4.2 Strengths and limitations

The Delphi method is an efficient means of reaching consensus.^8^ However, due to the complexity and scope of this subject area, this study benefitted from a careful preparatory process of systematic literature reviews of ambulance service measures and patient interviews to ensure a valid and comprehensive list of items was included. Each of the three consensus stages provided a key function: consensus event participants prioritized concepts and principles important to key stakeholder groups and members of the public; the Delphi process was used to develop and refine measures related to the consensus event findings; the PPI workshop allowed PPI representatives to engage and input with the prioritization process. We ensured participants represented a range of stakeholders from related disciplines to ensure that the results were relevant and meaningful to potential end users.

Common limitations of the Delphi process include selection bias in recruitment of participants and attrition over survey rounds.^27^ Our study recruited a range of expert participants and attrition was low. Many participants represented multiple participant categories; for example, all of the Emergency Care clinicians were also academics, as were several of the ambulance groups, meaning that this was a highly representative cohort of experts. There were more participants from ambulance‐related groups and PPI representatives because these groups will be most impacted by the research findings.

Our PPI colleagues identified that Delphi methods may not be appropriate for a lay audience when discussing complex medical or technical issues. Therefore, with our PPI reference group, we developed a suitable method to ensure meaningful PPI participation (a face‐to‐face workshop). However, this introduced some challenges. For example, it was not possible to include all measures from the Delphi in the PPI workshop. This was due to practical constraints regarding how many measures the PPI representatives could feasibly consider during a one day face‐to‐face event, given that each measure required substantial explanation and group discussion. There were also practical considerations relating to the amount of time PPI were able to contribute to the day, as well as travelling distances and potentially complex health problems to consider for participants. Therefore, measures were selected for inclusion in the PPI event based on the following criteria: those with the highest Delphi scores or related to categories of measures rated highly by Delphi participants, identified as important to PPI in our preliminary work or that our PPI reference group felt should be included. For example, when we considered pain measures, only one pain measure was highly scored by Delphi participants. However, this was identified as important by PPI representatives in our preliminary work, and our PPI reference group thought it was important that we include all the pain measures from the Delphi in the PPI consultation. Although we were unable to include all 61 measures from the Delphi study within the context of a one day PPI face‐to‐face event, we were able to obtain PPI views on a complex subject for the majority of measures from the Delphi study (35/61, 57%).

### 4.3 Value of PPI involvement

Including the views of PPI in meaningful rather than a tokenistic way is important, but was a challenging process. At times our planned research did not fit or suit a PPI audience and we had to be flexible in our research approach, as well as listening and responding to the views of our PPI reference group. In our study, an inflexible and unresponsive approach would have precluded significant PPI involvement. Without the efforts of our researchers and PPI reference group, who worked together to find a method that worked for the research study and the research participants, the voices of PPI would be missing. It is imperative that researchers value the role of PPI within a research study and work together to ensure meaningful involvement.

Some measures were considered at the PPI event despite achieving only moderate consensus from the Delphi study. These measures represented issues that were previously identified as important to PPI participants during our qualitative work or were considered as proxies for high scoring but difficult to measure items. Some high scoring measures were not considered at the PPI event. This was because it was felt they were already included or duplicated within very similar measures. For example, measures relating to time to definitive care at condition level included in the Delphi were presented as a single overall measure for the purposes of the PPI event.

Whole System measures were the highest scoring group of measures and included intermediate outcomes, organizational capability to respond to different types of calls and time‐related measures. Whole system measures may have scored highly because these represent the way in which people are accustomed to measuring the ambulance service and data are easily available.

### 4.4 Implications for research, policy and practice

Our programme of research (PhOEBE) sought to develop new quality measures for ambulance services. The findings of this study demonstrated consensus from a range of stakeholders for wider measures of ambulance quality that included structural, process and outcome measures of clinical effectiveness, patient safety and patient experience.^28^

The prioritized measures require further testing, developing and refining to facilitate their broader implementation. We also need to further understand how they might affect ambulance performance as well as any unintended consequences arising from their introduction.

Implementation also requires detailed specification of measures and, in some cases, modification to clinical records, processes and audit systems. Finally, some measures may require risk or case mix adjustment to facilitate benchmarking across services. Risk adjustment may also involve the development of predictive models to enable services to see the effects of population or service changes on care quality. The final stage of the project will involve building risk adjustment models for selected measures to inform the routine measurement of ambulance service performance in future.^29^

In summary, we have identified and prioritized a set of potential ambulance quality measures through a formal consensus process. The measures covered a broader range of domains than were currently used, relate to the whole ambulance service population and were identified as meaningful and important by a range of participants, including patients, public and ambulance services. The measures can be used to benchmark care quality between ambulance services or regions or to measure performance over time.

## CONFLICTS OF INTEREST

None.
