# Supplementary material for: Prioritizing novel and existing ambulance performance measures through expert and lay consensus: A three‐stage multimethod consensus study
Source: Health Expect. 2017 Aug 25;21(1):249–60. doi: 10.1111/hex.12610 (PMC5750751; doi:10.1111/hex.12610)
Supplement: Supplementary file 1 [file HEX-21-249-s001.docx]

Supplementary file 1: Outcome measures identified from the PhOEBE programme literature

reviews

**Direct clinical management of patients**

Calls sent for telephone nurse advice that are returned for an ambulance response

Accuracy of call taker identification of different conditions (e.g cardiac arrest, heart attack,

stroke, serious illness, low urgency calls suitable for nurse advice) or needs. Includes:

● measures of call assessment accuracy such as sensitivity

● appropriateness of triage decision

● risk of under-triage

● risk of over-triage

Accuracy of dispatch decisions – includes:

● Choice of response type dispatched (rapid response car, ambulance, helicopter)

● Appropriateness of referral to other agencies (e.g. GP services)

● Use of alternatives to ambulance dispatch (e.g. nurse advice or make own way)

● Relationship between priority category and response (right resource to right call)

Accuracy of paramedic diagnosis

● agreement of on-scene and final hospital diagnosis

● other measures of paramedic diagnosis accuracy, e.g. for specific conditions such as

stroke, trauma

Compliance with protocols and guidelines

● with triage protocols

● transport protocols (e.g leave at home, alternative to ED

● with care and treatment guidelines (fits and convulsions, heart attack, stroke

Proportion of people with respiratory distress (breathing difficulties) receiving

mechanically assisted breathing

Proportion of people with diabetes treated at home

Proportion of elderly people attended within scope of advanced paramedic practice (e.g.

treat and leave at home)

Proportion of people receiving spinal immobilisation (splints and collars) for back/neck

injuries

Re-contact with ambulance service within 24 hours (e.g. for calls closed with advice or

patients not transported)

Hospital attendance or admission (e.g. within 24 hours, 7 days, 28 days)

Re-admission within 30 days for complications (e.g pneumonia, wound infections)

Measuring patient safety

● Adverse incidents e.g. not recognising heart attack symptoms or leaving someone at

home who needed hospital treatment

● Errors in diagnosis

Length of stay in hospital

Duration of life support (intubation or ventilation) in hospital

Discharge destinations

● Home

● Continuing care

● Discharged needing continuing therapy e.g nursing care, supplemental oxygen, tube

feeding, assisted breathing

● Proportion of patients living at home at 3 months

Proportion of cases treated within time guidelines including:

● STEMI (heart attack) guidelines (90 minutes)

● Thrombolysis (clot busting) (60 minutes)

● Proportion FAST positive (suspected stroke) arriving at a stroke centre within 60

minutes

**Impact of care on patients**

Days lost from work following the emergency episode

Complications arising from care/treatment

● Pneumonia

● Wound infections

● Adverse drug effects (reactions)

Neurological (brain function) outcome at different time points (discharge, 1 month, 6

months, 1 year etc) using a variety of measures including:

● Glasgow Coma Score (adult and children)

● Glasgow Outcome score

● Cerebal Performance Category (CPC) (adult and children)

● Dementia score

Health/quality of life status

● Quality of life (EQ5D, SF-36)

● Function (Katz index of activities of daily living, Knauss class, McCabe Score, FIM

● Post traumatic stress disorder

Survival at different time points after the event:

● In hospital

● 30 days

● 90 days

● Six months

● 1 year

● 4-5 years

Patient experience

● Access

● Acceptability

● Decisions e.g. to leave at home

● satisfaction

● Professionalism

● Holistic care (physical, social, emotional needs)

Statistical methods for measuring survival

Pain measurement and symptom relief

● pain,

● nausea,

● Shortness of Breath (SOB)

● discomfort

Return of Spontaneous Circulation (ROSC) (return of pulse)

**Ambulance service activities and operations**

Call numbers and caller types

● Demographic (e.g age, gender) characteristics of service population

● Call volumes (numbers)

● Call volumes by incident types

● Geographical differences in use of emergency number

Call management characteristics (numbers and proportions)

● calls assigned to different urgency categories

● calls directed for nurse advice

● calls closed with nurse advice

● calls receiving paramedic response

● calls abandoned before answered

● ambulances cancelled

Utilisation (frequency of ambulance use)

● Utilisation by age groups/ethnic group/gender/poverty/incident types

● Utilisation per 1000 population

● Unit hour utilisation (use of resources)

Number of patients transported to hospital

● Transport rates for serious calls

● Transport rates for non-serious calls

Proximity of services

● % of operational area reachable within a specified time (e.g 10 mins, 20 mins, 30

mins, 1 hour

● % of population who can reach a major trauma centre within 45-60 minutes

● Scene to hospital distances

Proportion of calls treated by most appropriate service (whole 999 population)

Completeness and accuracy of patient records

Frequency with which ambulance staff administer treatments (e.g. inserting breathing

tubes, heart monitoring, oxygen therapy, defibrillation)

Service costs

● Cost per urgent call

● Cost per non-urgent call

● Cost per patient

● Mean cost of treatment (whole episode)

Ambulance service workforce characteristics

● Age - Average and proportions by group

● Attrition (staff turnover)

● Compensation claims

Types and numbers of patient transportations

● transport rates (numbers and proportions transported and not transported to

hospital)

● numbers and proportions transported to alternatives to Emergency department e.g.

minor injury unit

● numbers and proportions to different destination types (whole 999 population)

Ambulance staff training

● disability equality training

● communication skills

Volume and nature of complaints

Over – triage rates and under triage rates

● by category of urgency

● advice only

● condition specific e.g major trauma, stroke, STEMI (heart attack)

**Supplementary File 2** Delphi measures excluded from the PPI consensus workshop (supplementary online material)

| ID | **Whole system measures** | Delphi score | PPI Vote n (%) |
| --- | --- | --- | --- |
| R2_WS6e_3 | Proportion of eligible patients who arrive at a major trauma centre within 45 minutes | 9 | discussed under WS6e_1 |
| WS6e_2 | Proportion of eligible calls who arrive at a specialist stroke centre within 60 minutes | 9 | discussed under WS6e_1 |
| WS1a | Number of completed patient clinical records as a proportion of all cases attended by the ambulance service in accordance with minimum agreed dataset | 8 | N |
| WS6b | Proportion of emergency calls answered within 5 seconds | 8 | N |
| WS3a | Number of calls transferred for telephone clinical advice assessment that are completed with self-care advice or referral to an appropriate service as a proportion of call calls transferred for clinical advice | 7 | N |
| WS5b | Unit hour utilisation, urban areas (compared to agreed utilisation) | 7 | N |
| R2_WS6a_less_4 | Proportion of emergency calls for life threatening conditions with a response time of 4 minutes | 6 | N |
| R2_WS6a_4 | Proportion of emergency calls for life threatening conditions with a response time of less than 4 minutes | 6 | N |
| R2_WS6a_4_8 | Proportion of emergency calls for life threatening conditions with a response time of between 4 - 8 minutes | 6 | N |
| R2_WS6a_2_25mins | Proportion of emergency calls for conditions that are not life-threatening with a response time of 25 minutes or less | 6 | N |
| WS3d | Proportion of all calls who receive an ambulance response who are not conveyed to hospital/other health facility | 6 | N |
| WS6a_2 | Proportion of emergency calls with a response time within an agreed standard for non-life-threatening conditions | 6 | N |
|  | **Patient outcome measures** |  |  |
| PO5d | As PO5c but for specific clinical conditions (e.g stroke, heart attack, cardiac arrest) | 7 | N |
| PO6d2 | Proportion of patients left at home who are admitted to hospital within 72 hours | 6 | N |
| PO6d | Proportion of patients left at home who have a contact with any emergency/urgent health service within 72 hours | 5.5 | N |
| PO5b | Proportion of callers who died at different time points: specific groups e.g. condition, demographics, service | 6 | N |
| PO4a | Proportion of patients who have a wound treated at home who subsequently develop a wound infection | 6 | N |
